# Cocaine use in trauma patients admitted to intensive care medicine. clinical and epidemiological characterization

**DOI:** 10.1186/2197-425X-3-S1-A726

**Published:** 2015-10-01

**Authors:** F Verga, A Pascale, J Gil, A França, I Alvez, E Echavarría, H Bagnulo

**Affiliations:** Hospital Maciel, Intensive Care Unit, Montevideo, Uruguay; Hospital de Clínicas de Montevideo, Toxicology Department, Montevideo, Uruguay; Hospital de Clínicas de Montevideo, Statistics Department, Montevideo, Uruguay

## Introduction

Traumatic disease is a leading cause of death in younger patients. Substance abuse is a factor of risk for serious injuries and also influences the recurrence thereof. Detecting consumption is paramount to optimize the management of these patients in the care unit intensive as well as high once for prevention strategies secondary. Within these substances, the incidence of cocaine use in the South American cone is a growing health problem.

## Objectives

Determine the prevalence of cocaine use in severe trauma patients. Study the epidemiologic profile of this patients and compare clinical outcomes between cocaine users and non users.

## Methods

Prospective observational study conducted from January 2011 to December 2012.

## SETTING

Polyvalent Intensive Care Unit (ICU) of a tertiary hospital.

Patients: Severe trauma patients admitted to ICU during the study period.

Intervention: Determination of cocaine metabolites in urine.

Main measurements: Prevalence of cocaine use in severe trauma patients, demographic data, mechanical ventilation, complications, length of ICU stay and mortality.

## Results

302 patients were included, 82,5% males, age (median ± interquartile range) 34 ± 27 years, Simplified Acute Physiology Score (SAPS II) 35 ± 26, mortality 17,2%. Cocaine use was detected in 82 cases (27,2%). Positive cocaine screening pacients were younger (30 ± 13 vs 37 ± 31; p < 0,001) y mostly males (29,7% vs 15,1%; p = 0.03). No significant differences were found about mortality, need or duration of mechanical ventilation and length of ICU stay. A significant association between positive cocaine screening and agitated awakening (46,3% vs 30,9%, p = 0,035), extubation failure (15,6% vs 6,0%, p = 0,019) and late onset pneumonia (48,9% vs 29,5 %, p = 0,020) was found.

## Conclusions

A high prevalence of cocaine use in trauma patients who were admitted to ICU was found in comparison with international reports. Epidemiological profile includes young males cocaine users with a higher incidence of agitated awakening, extubation failure and late onset pneumonia.
